# Anxiolytic and Antidepressant Effects of *Tribulus terrestris* Ethanolic Extract in Scopolamine-Induced Amnesia in Zebrafish: Supported by Molecular Docking Investigation Targeting Monoamine Oxidase A

**DOI:** 10.3390/ph17091208

**Published:** 2024-09-13

**Authors:** Salwa Bouabdallah, Mona H. Ibrahim, Ion Brinza, Razvan Stefan Boiangiu, Iasmina Honceriu, Amr Amin, Mossadok Ben-Attia, Lucian Hritcu

**Affiliations:** 1 Environmental Biomonitoring Laboratory, Bizerte Faculty of Sciences, Carthage University, Zarzouna 7021, Tunisia; 2 Department of Biology, Faculty of Biology, Alexandru Ioan Cuza University of Iasi, 700506 Iasi, Romania; 3 Department of Pharmaceutical Medicinal Chemistry and Drug Design, Faculty of Pharmacy (Girls), Al-Azha University, Cairo 11884, Egypt; 4 College of Medicine, University of Sharjah, Sharjah 27272, United Arab Emirates

**Keywords:** Alzheimer’s disease, depression, kaempferol, locomotor activity, monoamine oxidase A, NTT

## Abstract

Plants of the genus *Tribulus* have been used in folk medicine for wound healing, alleviating liver, stomach, and rheumatism pains, and as cognitive enhancers, sedatives, antiseptics, tonics, and stimulants. The present work aimed to evaluate whether *Tribulus terrestris* (Tt) administered for 15 days attenuated cognitive deficits and exhibited anxiolytic and antidepressant profiles in scopolamine-induced amnesia in zebrafish. Animals were randomly divided into six groups (eight animals per group): (1)–(3) Tt treatment groups (1, 3 and 6 mg/L), (4) control, (5) scopolamine (SCOP, 0.7 mg/kg), and (6) galantamine (Gal, 1 mg/L). Exposure to SCOP (100 µM) resulted in anxiety in zebrafish, as assessed by the novel tank diving test (NTT) and novel approach test (NAT). When zebrafish were given SCOP and simultaneously given Tt (1, 3, and 6 mg/L once daily for 10 days), the deficits were averted. Molecular interactions of chemical compounds from the Tt fractions with the monoamine oxidase A (MAO-A) were investigated via molecular docking experiments. Using behavioral experiments, we showed that administration of Tt induces significant anxiolytic-antidepressant-like effects in SCOP-treated zebrafish. Our result indicated that flavonoids of Tt, namely kaempferol, quercetin, luteolin, apigetrin, and epigallocatechin, could act as promising phytopharmaceuticals for improving anxiety-related disorders.

## 1. Introduction

Anxiety and depression have been shown to intensify the severity of cognitive decline in Alzheimer’s disease [1]. Furthermore, individuals with dementia experience anxiety more frequently than those without dementia [2]. It is linked to problematic behaviors, reduced quality of life, difficulties in performing daily activities, night-time awakenings, and decreased neuropsychological performance, even when depression is controlled [3]. The World Health Organization (WHO) estimates that around 350 million people suffer from depression and expects that by 2020, the condition will be the second leading cause of disability worldwide. Despite the availability of numerous effective antidepressants, many cause inadequate and disappointing results in about one-third of all treated subjects [4].

Zebrafish (*Danio rerio*) have recently emerged as a relevant experimental species for investigating normal and dysfunctional biological processes. This is due to the sequencing of their genome, their short generational span, their high reproductive rate, and their ability to be housed at high densities compared to laboratory mammals [5,6,7,8,9,10]. Kalueff and collaborators have highlighted the relevance of zebrafish as a valid experimental tool for closely examining the biological determinants of behavior [11]. Consequently, zebrafish have been used to probe the fundamental mechanisms underlying individual responses to addictive drugs, emotional models of exposure, and higher-order brain functions [12].

Several behavioral paradigms can be similarly applied to both mammals and zebrafish [13,14,15]. The novel tank test (NTT) is notably effective for assessing anxiety, with some translational relevance to humans [16,17,18]. This behavioral test capitalises on the natural tendency of zebrafish to dive and remain at the bottom of a water tank to avoid perceived danger or stress. The scientific literature supports the use of zebrafish tests to investigate anxiety-like states and anxiolytic-like effects of plant secondary metabolites, such as flavonoids, saponins, alkaloids, and terpenes or standardized extracts of other plants, which represent a valuable and ethical tool in the initial stages of behavioral research [19,20].

Phytotherapies may be an interesting and successful option in the remedy of depression, as a significant number of herbal preparations have shown psychotherapeutic activities.

The discovery of new pharmacotherapy from medicinal plants and their isolated constituents for psychiatric disorders, including depression, has advanced significantly over the past decade [21,22,23]. Many flavonoids exhibit antidepressant and antioxidant activities [24,25,26,27]. It is widely recognised that oxidative stress plays a critical role in the development of various diseases, including psychopharmacological dysfunction [28]. Indeed, the association between oxidative stress and depression has been extensively discussed and analyzed in several reviews [29].

The genus *Tribulus*, belonging to the family Zygophyllaceae, includes around 20 species globally [5]. In India, three species are commonly found: *Tribulus terrestris* (Tt), *Tribulus cistoides*, and *Tribulus alatus*. Among these, Tt is widely distributed across North Africa [6]. Tt is a popular medicinal plant widely used in India and other East Asian countries. It is well known for its aphrodisiac effects in Ayurvedic medicine. Traditionally, it has been employed as a restorative agent for abdominal distension, sexual dysfunction, leucorrhea, eye disorders, asthma, hyperlipidemia, hypertension, microbial infections, and clouding [30]. Numerous studies have demonstrated its pharmacological potential, including aphrodisiac and fortifying effects [31], hypoglycemic, diuretic [32], antihemorrhagic, astringent, tonic [33], anticancer [33], antileishmanial properties [34] and neuroprotective effects [35].

Monoamine oxidase (MAO) inhibitors are naturally derived compounds that have been developed clinically for the treatment of depression, social anxiety, and Parkinson’s disease [36,37,38,39,40]. Monoamine oxidase is a widely distributed mitochondrial enzyme, highly expressed in the brain as well as in gastrointestinal and hepatic tissues [41].

Recent research is frequently dedicated to enhancing our understanding of the role of monoamine oxidase A’s (MAO-A) in regulating anxiety and developing more precise pharmacological strategies [42]. Investigations explore new drug candidates, mechanisms of action, and potential side effects or interactions with other medications. MAO-A is an enzyme that plays a key role in the metabolism of neurotransmitters in both the central nervous system (CNS) and peripheral tissues [41]. Serotonin, norepinephrine, and dopamine are neurotransmitters directly involved in the regulation of mood and emotions. MAO-A inhibitors are a class of drugs historically used to treat anxiety disorders and depression. They have been reported to alleviate depression and specific types of anxiety [41]. These drugs work by blocking the activity of MAO-A, thereby increasing the levels of neurotransmitters such as serotonin, norepinephrine, and dopamine in the brain. This elevation in neurotransmitter levels is believed to contribute to their therapeutic effects in relieving symptoms of anxiety and depression. Targeting MAO-A is thus relevant for treating anxiety, and MAO inhibitors have the potential to be used as therapeutic medicines, particularly for conditions characterized by excessive MAO enzyme expression.

To date, no studies in the literature have explored the anxiolytic and antidepressant effect of Tt in a zebrafish model targeting MAO-A. This study conducts a thorough investigation into the anxiety-reducing effect of polyphenols and their interactions with oxidoreductase enzymes. Furthermore, it incorporates computational analysis to examine the interactions of kaempferol, quercetin, luteolin, apigetrin, and epigallocatechin with MAO-A, intending to assess their potential for anxiolytic effects in vivo.

## 2. Results

### 2.1. In Vivo Bioassays

In vivo studies evaluated the anxiety-like behaviors exerted by Tt, in a model of SCOP-induced cognitive deficits and an increase in anxiety levels in zebrafish (*Danio rerio*). Both the novel tank dividing test (NTT) and novel approach test (NAT) were employed.

### 2.2. Effects on the Zebrafish NTT Response

After administering Tt at concentrations of 1, 3, and 6 mg/L, which were selected based on our previous work [35], zebrafish exhibited no behavioral abnormalities, toxicity, or mortality, indicating that these dosages of Tt are safe.

**Figure 1:**
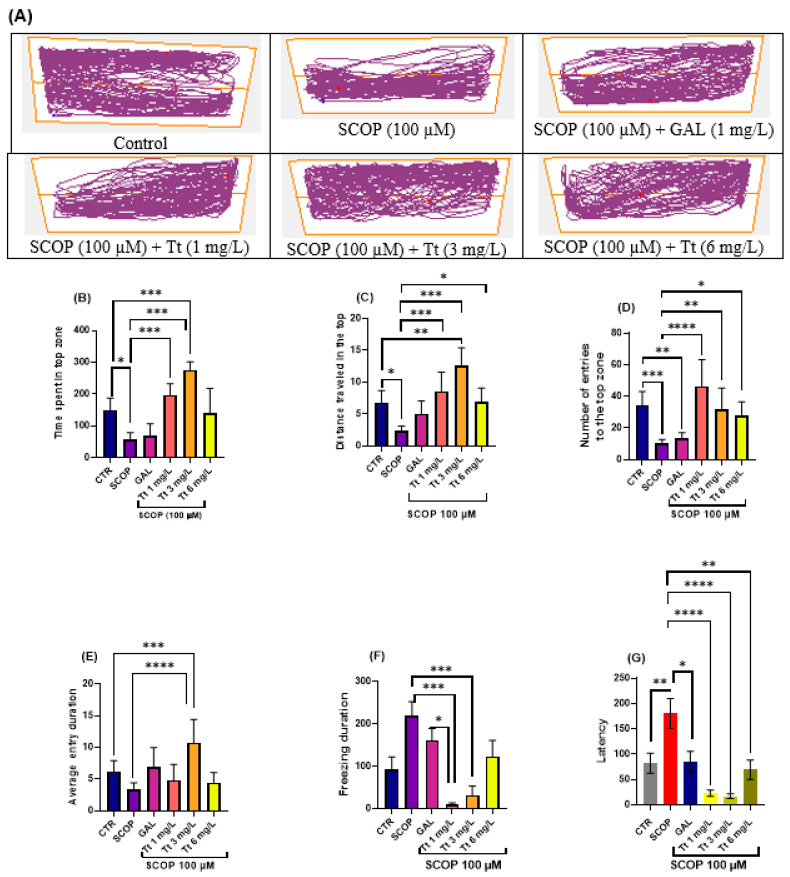
Novel tank dividing test (NTT) results for *Tribulus terrestris* (Tt: 1, 3 and 6 mg/L). (**A**) Representative tracking locomotion patterns; (**B**) Time spent in the top (s); (**C**) Distance travelled in the top (m); (**D**) Number of entries to the top (s); (**E**) Average entry duration; (**F**) Freezing duration (s); (**G**) Latency. Data are expressed as means ± S.E.M. (*n* = 8). * *p* < 0.01, ** *p* < 0.001, *** *p* < 0.0001, and **** *p* < 0.00001 (Tukey’s post hoc analyses). Galantamine (GAL, 1 mg/L) was used as a reference positive drug.

The effects of SCOP (100 µM) and Tt (1, 3, and 6 mg/L) on anxiety-like behavior in the NTT test are depicted in Figure 1. In the NTT, the typical locomotion tracking patterns (Figure 1A) display the differences in swimming traces between the top and bottom zones. SCOP treatment significantly decreased the time spent in the top zone of the tank (*p* < 0.001) as compared with the control group, suggesting a SCOP-induced anxiogenic effect (Figure 1B). Additionally, SCOP treatment induced a hypolocomotor effect compared to the control group, observed as a decrease in the distance top/bottom ratio (*p* < 0.01) (Figure 1C). One-way ANOVA revealed a significant effect of the Tt (1, 3, and 6 mg/L) treatment on the time spent in the top zone of the tank (F (8, 63) = 13.24, *p* < 0.0001) (Figure 1B), on distance top/bottom ratio (F (8, 63) = 9.56, *p* < 0.0001), on the number of entries to the top of the tank in the NTT (F (8, 63) =8.40 *p* < 0.0001) (Figure 1D). In addition, one-way ANOVA revealed significant overall differences in the time spent in the top (F (4, 45) = 13.24, *p* < 0.0001) (Figure 1B), the average entry duration (F (8, 63) = 6.64, *p* < 0.0001) (Figure 1E), the distance travelled in the top (F (8, 63) = 9.56, *p* < 0.0001), the total distance travelled (F (4, 45) = 5.00, *p* < 0.0001), the freezing duration F (8, 63) = 4.698, *p* = 0.0002 (Figure 1F) and in latency F (8, 61) = 8.13, *p* < 0.0001) (Figure 1G). Moreover, treatment with the tested fractions and compounds of Tt (1, 3, and 6 mg/L) prevented the hypolocomotor effect of SCOP on the velocity (*p* < 0.001) as compared with SCOP-alone-treated fish. An increase in the distance top/bottom ratio was also observed for Tt, (*p* < 0.001) for 1 and 3 mg/L). In Tt, the anxiolytic-like effect was noticed by increasing the time spent in the top zone of the tank (*p* < 0.0001) as compared with the SCOP-alone treated animals. Overall, Tt showed anxiolytic effects.

### 2.3. Effects on Zebrafish Novel Approach Test Response

**Figure 2:**
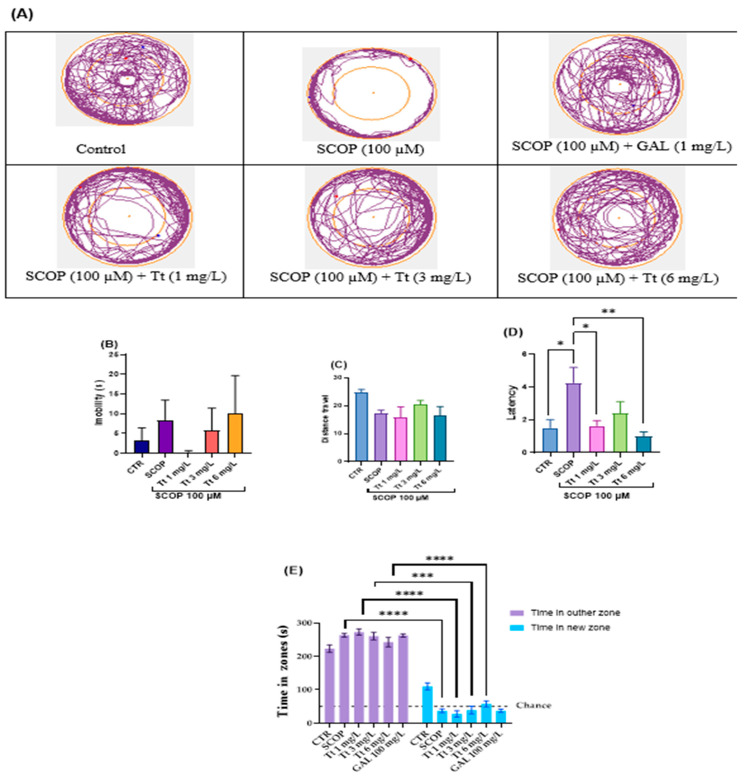
Novel approach test (NAT) results for *Tribulus terrestris* (Tt: 1, 3, and 6 mg/L). (**A**) Representative tracking locomotion patterns; (**B**) Immobility (s); (**C**) Distance travelled (m); (**D**) latency; (**E**) Times in zones (s). Data are expressed as means ± S.E.M. (*n* = 8). * *p* < 0.01, ** *p* < 0.001, *** *p* < 0.0001, and **** *p* < 0.00001 (Tukey’s post hoc analyses). Galantamine (GAL, 1 mg/L) was used as a reference positive drug.

Moreover, the anxiety-like behavior was evaluated in the novel approach test (NAT) as is depicted in Figure 2. In Figure 2A, the variations in the locomotor tracking behaviors of distinct groups of zebrafish in the inner and the outer zones of the tank were noticed. SCOP had significant effects on locomotion (immobility and distance travelled) as shown in (Figure 2B,C). Yet, such latency varied depending on the experimental group: One-way ANOVA of the immobility F (7, 56) = 0.78, *p* < 0.5 (Figure 2B), distance travelled: F (7, 56) = 2.29, *p* < 0.05 (Figure 2C), and latency (F (7, 56) = 3.21, *p* < 0.01 (Figure 2D). Tukey’s test analysis of time in the inner zone for Tt (1, 3 and 6 mg/L), (*p* < 0.0001) compared to the control group in the outer zone (Figure 2E).

### 2.4. Molecular Docking Analysis

**Figure 3:**
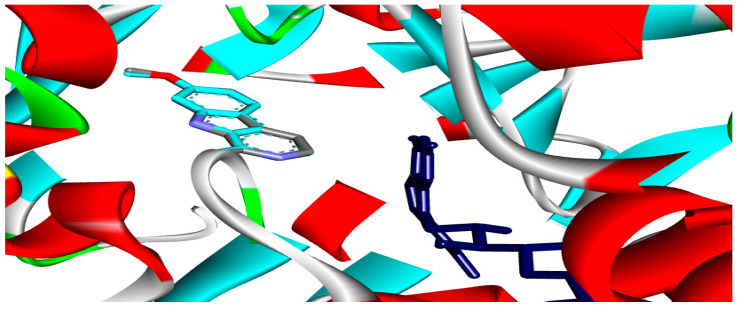
The root means square deviation (RMSD) between the original and docked poses of the co-crystal ligands for the MAO-A enzyme (PDB: 2z5x) was 0.13 Å.

Six flavonoid compounds—rutin, apigetrin, kaempferol, quercetin, luteolin, and epigallocatechin, along with six saponin compounds-saponin C, protodioscin, terrestrosin C, trillarin, terreside B, and disogluside were subjected to docking against the MAO-A enzyme. The flavonoid ingredients kaempferol, quercetin, luteolin, apigetrin, and epigallocatechin exhibited significant interactions, as demonstrated in Table 1. However, rutin and the saponin compounds showed no activity against the MAO-A enzyme. The validity of the docking approach was validated by re-docking the co-crystallized ligand, which resulted in a low Root Mean Square Deviation (RMSD) of 0.13 Å (Figure 3). The docking scores for the five flavonoid compounds varied between −7.2 and −9.7 kcal/mol.

## 3. Discussion

The findings of this study indicated that Tt, recognized for its anti-cancer, anti-inflammatory, and neuroprotective properties [34,35,36], enhanced behavioral outcomes in an experimental model of SCOP-induced depression and anxiety. These effects were dose-dependent and exhibited anxiolytic-like properties.

Our present work aims to determine if *Tribulus* extracts have a potential anxiolytic activity in a zebrafish model with dementia-like conditions such as Alzheimer’s disease (AD). SCOP was used to induce dementia-like AD, and it was found that SCOP (1 mg/kg) can impair memory consolidation in the hippocampus, following the method of Zaki et al. [43].

Anxiety is a pervasive trait found across many perceptual disorders, and understanding its fundamental biology could significantly contribute to the development of new pharmacotherapies [2,40]. To address this issue, zebrafish have been extensively used in the translational neuroscience of affective disorders with anxiety being a primary focus of exploration [1,2,43].

The two most commonly used assays for assessing anxiety-like behavior in zebrafish are the light–dark test (LDT) and the novel tank diving test (NTT) [4]. The NTT and LDT have been widely validated using drugs that produce anxiogenic and anxiolytic effects across species, including humans [44,45,46,47]. Various types of anxiety disorders are recognized in the Diagnostic and Statistical Manual of Mental Disorders by the American Psychiatric Association, such as generalized anxiety disorder, panic disorder, social anxiety disorder, and agoraphobia [47,48]. The zebrafish NTT closely resembles agoraphobia, a type of panic disorder where individuals experience anxiety in environments perceived as unsafe with no easy escape. In this test, zebrafish exhibit anxiety-like behavior by avoiding potential threats, such as predators, in a new environment, often by diving to the tank’s bottom [48].

Kafeel and Rukh [49] evaluated the anxiolytic potential of Tt ethanolic extract in experimental mice using the Light-Dark Box (LDB), Elevated Plus Maze (EPM), and Head Dip models of anxiety. In the LDB model, EETT demonstrated an increase in both the number of entries and the time spent in the light compartment. Additionally, the time spent in the open arms of the EPM was significantly increased *p* < 0.05 in comparison with a control group in EPM apparatus. An increase in the number of head dips is also suggestive of the possible anxiolytic potential of Tt.

Traditionally, MAO inhibitors have been considered for treating panic disorders with agoraphobia, albeit their use has been limited due to potential risks like hypertension. However, the flavonoids investigated in this study are reversible MAO inhibitors, preferred for their lower incidence of side effects [50,51].

The UPLC-EIS/MS analysis of Tt was developed as described in our recent publication [36] in Table 2. Our results revealed the presence of various flavonoids and saponins, including epigallocatechin, kampeferol, rutin, quercetin, luteoline, apigetrin cynaroside, caffeic acid, trillin, trillarin, hecogenin, terreside B, protodioscin, and saponin C [35]. We aimed to study the interaction between Tt compounds and MAO-A. To accomplish this, we selected 12 compounds identified in Tt: 6 flavonoids and 6 saponins as mentioned in Table 2.

Kaempferol and isorhamnetin have been demonstrated to mitigate LPS-induced anxiety and depression in various tests, including the Open Field Test (OFT), LDT, Elevated Plus Maze (EPM), Forced Swim Test (FST), and Tail Suspension Test (TST) as reported by Hashemzaei et al. [37]. These flavonoids reduced oxidative stress in the prefrontal cortex and hippocampus by decreasing malondialdehyde (MDA) and total oxidant system (TOS) levels while increasing total antioxidant status (TAS) levels. They also attenuate LPS-induced inflammation by decreasing reducing levels of TNF-α, IL-1β, and IL-6. Additionally, LPS decreased Brain-Derived Neurotrophic Factor (BDNF) levels in these brain regions, which was reversed by Kaempferol and Isorhamnetin. These findings underscore the potential therapeutic benefits of Kaempferol and Isorhamnetin and highlight the roles of oxidative stress, inflammation, and BDNF in the development of anxiety and depression. Furthermore, quercetin has been reported to possess various biological effects, including antioxidant [29], anti-inflammatory [38], anxiolytic [39], and neuroprotective ones [37]. Cumulative reports suggest that quercetin exhibits anxiolytic-like effects in experimental animals [39]. Studies indicate that quercetin may interact with the GABA-α5 receptor to relieve seizures and with the GABA receptor β1 and β3 subunits for its anti-epileptic effect [42].

For many years, the hippocampus has been recognized for its role in regulating learning and memory abilities, as well as its significant involvement in mood regulation. Research findings suggest that the hippocampus also acts as a gateway to the prefrontal cortex, a critical brain region involved in stress and stress-related behaviors. The prefrontal cortex plays a crucial role in working memory, executive function, self-regulatory behaviors, and stress response [52,53]. Both of these brain regions are profoundly affected by numerous psychiatric disorders, particularly anxiety and depression [54]. In this study, it was found that MDA and TOS levels increased while TAS levels decreased in the hippocampus and prefrontal cortex following flavonoid administration. This indicates that flavonoids induce oxidative stress in these brain areas.

**Figure 4:**
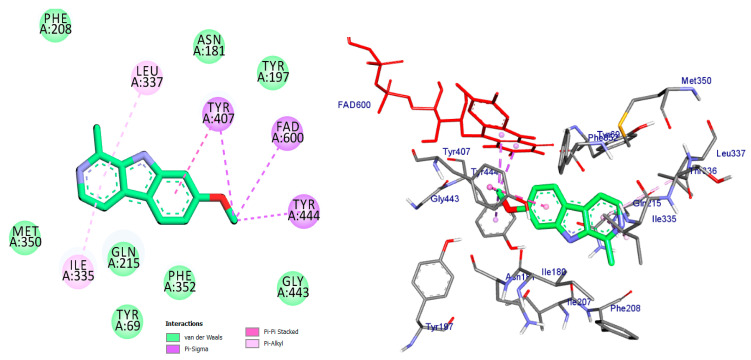
2D and 3D representation of co-crystal ligand docked into binding site of MAO_A active site enzyme.

**Figure 5:**
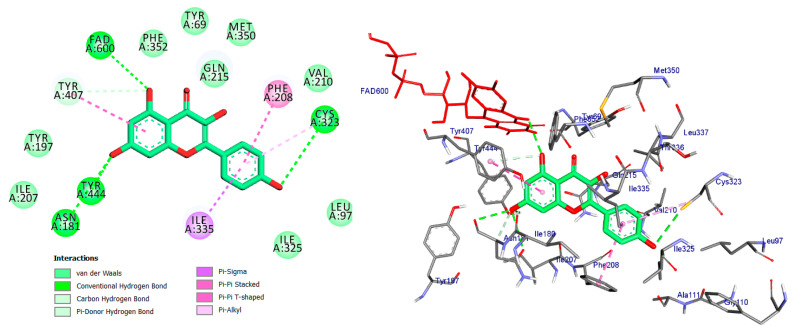
2D and 3D representations of Kaempferol docked into binding site of MAO-A active site enzyme.

**Figure 6:**
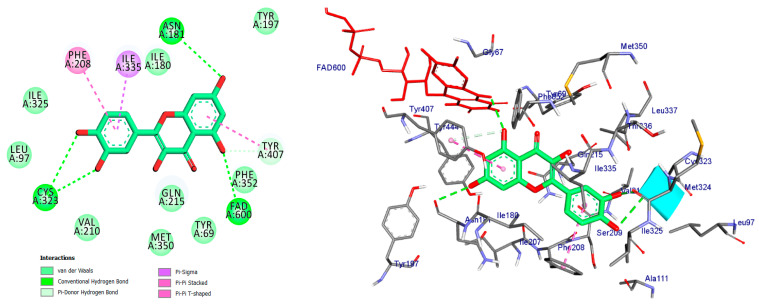
2D and 3D representations of Quercetin docked into binding site of MAO-A active site enzyme.

**Figure 7:**
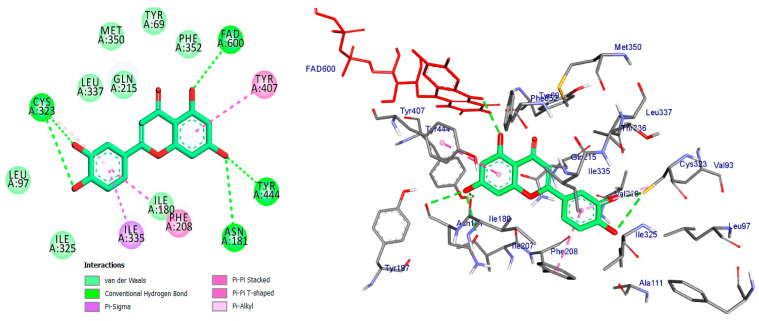
2D and 3D of Luteoline docked into binding site of MAO-A active site enzyme.

Monoamine oxidases (MAOs) are widely distributed enzymes that contain a flavin adenine dinucleotide (FAD) cofactor chemically attached to a cysteine residue. These enzymes are present in multiple living organisms, with mammals having two specific types known as MAO-A and MAO-B. These isoforms play a crucial role in the metabolism of key neurotransmitters in both the central nervous system (CNS) and peripheral tissues [41]. Blocking MAOs can lead to increased levels of neurotransmitters such as serotonin and dopamine, which are stored in nerve terminals. Therefore, MAO inhibitors have the potential to be used as therapeutic agents, particularly for medical conditions characterized by excessive MAO enzyme expression. The human MAO-A enzyme features a single substrate cavity [40]. Docking analysis revealed that the co-crystal ligand (Harmine), a reversible inhibitor, binds within the enzyme’s active center cavity. This interaction involves Pi-sigma, Pi-alkyl, or Pi-Pi stacked interactions with Tyr-407, Tyr-444, FAD-600, Leu-337, and Ile-325, as depicted in Figure 4 and Table 1. The constituents of the five flavonoids showed favorable binding to the MAO-A enzyme. All of them interacted with critical residues such as Tyr-407, FAD, Ile-335, and Cys-323 (Table 1). The substances that exhibited the highest activity were kaempferol, quercetin, and luteoline, with binding energies of −9.7, −8.7, and −8.8 kcal/mol, respectively. In comparison, Harmine had a binding energy of −8.7 kcal/mol. The chromen moiety of these inhibitors formed a hydrogen bond with the cofactor FAD and a hydrophobic interaction with Tyr-407 (Figure 5, Figure 6 and Figure 7).

The compound kaempferol exhibited the highest level of inhibition and formed five typical hydrogen bonds with Cys-323 (at a distance of 3.53 Å), Tyr-444 (at a distance of 2.45 Å), FAD-600 (at a distance of 2.95 Å), and Asn-181 (at distances of 3.31 Å and 3.18 Å). Additionally, it established four hydrophobic contacts with Ile-335, Tyr-407, Phe-208, and Cys-323. Furthermore, kaempferol formed a carbon–hydrogen bond with Asn-181 and a pi-donor hydrogen bond with Tyr-407 (Figure 5, Table 1).

Quercetin formed four traditional hydrogen bonds with Cys-323 (at distances of 3.04 and 3.70 angstroms), FAD (at a distance of 2.70 angstroms), and Asn-181 (at a distance of 3.30 angstroms). Additionally, quercetin demonstrated four pi interactions with Tyr-407, Ile-335, and Phe-208 as depicted in Figure 6 and summarized in Table 1.

Finally, the luteolin molecule formed six hydrogen bonds with Tyr-444, Cys-323, FAD-600, Thr-336, Phe-208, and Asn-181. It also formed a pi-donor hydrogen bond with Tyr-407 and Tyr-444, along with four hydrophobic interactions (Figure 7 and Table 1).

For decades, the hippocampus has been recognized for its pivotal role in regulating learning, memory abilities, and mood regulation [40,41]. Research highlights the hippocampus as a conduit to the prefrontal cortex, a critical brain region involved in stress responses and related behaviors, such as working memory, executive function, and self-regulation [53,54]. These brain regions are significantly implicated in various psychiatric disorders, notably anxiety and depression [24]. This study revealed that following the administration of flavonoids, the levels of malondialdehyde (MDA) and total oxidant status (TOS) increased, while the total antioxidant status (TAS) levels decreased in both the hippocampus and prefrontal cortex. These changes suggest that flavonoids induce oxidative stress in these brain areas.

In our recent publication, we explored the role of flavonoids in Alzheimer’s disease and conducted docking simulations with the acetylcholinesterase enzyme [55]. The four selected phytoconstituents demonstrated favorable binding affinities ranging from −6.64 to −7.50 kcal/mol in the docking simulation. The results suggest that these molecules possess potential anti-acetylcholinesterase activity with low toxicity. Moreover, ADME analysis indicated that they have high absorbability into the bloodstream [55].

In this work, we selected MAO-A as the drug target due to its crucial role in neurotransmitter metabolism in both the central nervous system (CNS) and peripheral tissues. We performed docking simulations on six flavonoid molecules (rutin, apigetrin, kaempferol, quercetin, luteolin, and epigallocatechin) and six saponin molecules (saponin C, protodioscin, terrestrosin C, trillarin, terreside B, and disogluside). Of these, five flavonoids showed significant interactions with MAO-A, while rutin and all six saponin molecules failed to bind to the enzyme. This highlights the need for further investigation and opens new avenues for future research discussions.

## 4. Materials and Methods

### 4.1. Plant Material

*Tribulus terrestris* (Tt) was collected from Tunisia’s native flora. The plants were found growing wild in the Al-Hawaria area. The specimens were collected at the mature fruit phase in September 2020, and identified as mentioned in our last publication [33]. A series of specimens was stored in the Environmental Biomonitoring Laboratory LBE (LR01/ES14), University of Carthage, Tunisia. Leaves, seeds, and roots were carefully separated, and the samples were kept at 4–5 °C in Enfield plastic containers, in batches of 500 g of each part.

### 4.2. Conventional Heat Reflux Extraction

The dried and powdered plant material (50 g) was sequentially extracted first with chloroform at room temperature (3 × 450 mL for 1 h each) and then with 70% ethanol using Soxhlet reflux at 80 °C (3 × 450 mL for 2 h each). The combined ethanol extracts were concentrated under vacuum at 70 °C to obtain a final extract (The extraction yield is approximately 33%).

### 4.3. Fish Care and Maintenance

Wild-type zebrafish (*Danio rerio*; short-fin strain) of both sexes (50:50 ratio) were housed in the animal facility within Alexandru Ioan Cuza University of Iasi, Faculty of Biology, Romania following standard procedures (Zebrafish Information Network) as described previously [35]. Briefly, zebrafish were housed in three 70-L aquariums within a recirculation system that supplied well-ventilated and dechlorinated water maintained at a controlled temperature of 26 °C ± 2. The photoperiod was set to a 14-h light to 10-h dark cycle. Water quality parameters were consistently maintained: pH at 7.5, dissolved oxygen at 7.20 mg/L, ammonium concentration below 0.004 ppm, and conductivity at 500 µS.

### 4.4. Animals and Drug Treatment

**Figure 8:**
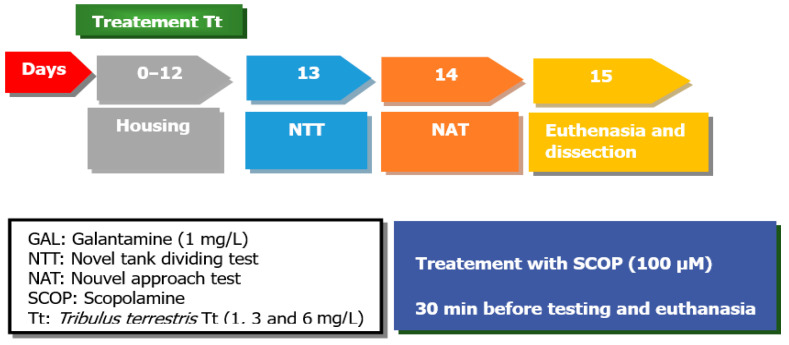
The experimental design of the study (NTT and NAT test).

All animals (48) were divided into the following groups: Tt was dissolved in DMSO (1%) at concentrations of 1, 3, and 6 mg/L (three Tt pre-treatment groups), the control group, the SCOP group (SCOP, 100 µM, Sigma-Aldrich, Darmstadt, Germany), and the galantamine group (GAL, 1 mg/L, Sigma-Aldrich, Darmstadt, Germany), as a positive control, within the NTT test and NAT test. The doses of SCOP and GAL were carried out as reported in the previous study [35]. Tt (1, 3, and 6 mg/L) was administered by immersion once daily into a 6 L glass for 1 h, as well as SCOP (100 µM) 30 min before starting the behavioral tests (Figure 8).

### 4.5. Behavioral Evaluation

A Logitech HD Webcam C922 Pro Stream camera (Logitech, Lausanne, Switzerland) was used to record zebrafish behavior. The recorded videos were analyzed using ANY-maze^®^ software version 7.4 (Stoelting Co., Wood Dale, IL, USA).

### 4.6. Novel Tank Diving Test (NTT)

The NTT is a particular test used to evaluate both locomotor activity and anxious responses in zebrafish as noted by Cachat et al. [1]. A 1.5 L trapezoidal tank (15.2 × 27.9 × 7.1 cm) was divided by a virtual horizontal line into top and bottom sections. Zebrafish were individually tested for 6 min and analyzed using ANY-maze^®^software 7.4 (Stoelting Co., Wood Dale, IL, USA). The locomotor activity endpoints consisted of the total distance travelled (m) and average velocity (m/s) and the anxiety-like behavior was estimated by the number of entries to the top, time spent in the top (s), average entry duration (s), and freezing duration (s).

### 4.7. Novel Approach Test (NAT Test)

The experiment was conducted in a safe plastic arena with a diameter of 34 cm, a circumference of 108.5 cm, and a depth of 15 cm, following previous descriptions [56]. Positioned at the center of the arena was a LEGO^®^ figurine measuring 2 cm × 4.25 cm, designed with multiple colours to prevent potential influences from inherent colour preferences [57,58]. The behavior of individual zebrafish for 5 min was analyzed by ANY-maze⁠^®^software 7.4 (Stoelting Co., Wood Dale, IL, USA). The arena’s configuration in ANY-maze encompassed an inner and outer zone, marked by a cantered virtual circle with a diameter of 17 cm. The duration zebrafish spent in each zone was recorded in seconds, and locomotor activity was measured in terms of the distance travelled (m) and immobility (s).

### 4.8. In Silico Docking Experiments

The Protein Data Bank supplied the crystal structures of human monoamine oxidase A, identified by the PDB ID: 2z5x [59]. The docking procedures were executed via Autodock Vina 4.2 [60], requiring the receptor and ligands to be in pdbqt format. Prior to docking, it was imperative to utilize M.G.L tools to generate the two enzymes, the co-crystalized substance, and the flavonoid and saponin components in the suitable configuration [61]. The outcomes of the docking process were exhibited utilizing the Discovery Studio 4.5 visualizer (D.S. BIOVIA, San Diego, CA, USA; 2005) [62]. A 3D grid box of 50 × 50 × 50 Å (*x*, *y*, *z*) with a spacing of 0.375 Å is located at coordinates 40.58, 26.93, and −14.54 Å for the purpose of docking into human monoamine oxidase A.

### 4.9. Statistical Analysis

All results are expressed as mean ± standard error of the mean (S.E.M) and were analyzed by GraphPad Prism 9.0 software (GraphPad Software, Inc., San Diego, CA, USA). Datasets with multiple comparisons were evaluated using one-way or two-way ANOVA followed by Tukey’s post hoc test. *p* < 0.05 was considered to show a statistically significant difference.

## 5. Conclusions

For the first time, behavioral tests revealed that Tribulus terrestris (Tt) effectively reduced scopolamine (SCOP)-induced anxiety and cognitive deficits in a zebrafish model, highlighting its potential as an anxiolytic agent. Additionally, kaempferol, quercetin, and luteolin showed significant interactions with the MAO-A enzyme, suggesting potential benefits for promoting neurogenesis.

## Figures and Tables

**Table 1:** Types of interactions, Amino acids involved, distance, and binding energy of Co-crystal ligand (Harmine), Apigetrin, Kaempferol, Quercetin, Luteoline, and Epigallocatechin within MAO-A enzyme active site.

Compound Name	Interaction/Amino Acid/Distance Å	Docking Energy Scores in kcal/mol
Co-crystal ligand (Harmine)	Pi-Sigma/Tyr407/3.74 Pi-Sigma/Tyr444/3.65 Pi-Sigma/FAD600/3.66 Pi-Sigma/FAD600/3.72 Pi-Pi Stacked/Tyr407/4.29 Pi-Alkyl/Ile335/4.46 Pi-Alkyl/Leu337/5.40	−8.7
Apigetrin	Conv. H-Bond/Val210/2.11 Conv. H-Bond/Cys321/3.43 Conv. H-Bond/Cys323/3.74 Conv. H-Bond/Thr336/2.57 Conv. H-Bond/Ala111/2.71 Car H-Bond/Ser209/3.42 Pi-Donor H-Bond/Tyr407/3.57 Pi-Donor H-Bond/Tyr444/3.90 Pi-Donor H-Bond/FAD600/4.05 Pi-Donor H-Bond/FAD600/4.12 Pi-Sigma/Ile335/3.50 Pi-Sulfur/Cys323/4.93 Pi-Pi Stacked/Tyr407/4.29 Pi-Alkyl/Leu337/5.23	−7.2
Kaempferol	Conv. H-Bond/Cys323/3.53 Conv. H-Bond/Tyr444/2.45 Conv. H-Bond/FAD600/2.95 Conv. H-Bond/Asn181/3.31 Conv. H-Bond/Asn181/3.18 Car H-Bond/Asn181/3.58 Pi-Donor H-Bond/Tyr407/4.12 Pi-Sigma/Ile335/3.54 Pi-Pi Stacked/Tyr407/4.78 Pi-Pi T-shaped/Phe208/4.71 Pi-Alkyl/Cys323/5.30	−9.7
Quercetin	Conv. H-Bond/Cys323/3.04 Conv. H-Bond/Cys323/3.70 Conv. H-Bond/FAD600/2.70 Conv. H-Bond/Asn181/3.30 Pi-Donor H-Bond/Tyr407/4.16 Pi-Sigma/Ile335/3.51 Pi-Pi Stacked/Tyr407/4.59 Pi-Pi T-shaped/Phe208/4.60	−8.7
Luteoline	Conv. H-Bond/Cys323/3.00 Conv. H-Bond/Cys323/3.55 Conv. H-Bond/Tyr444/2.47 Conv. H-Bond/FAD600/3.01 Conv. H-Bond/Asn181/3.38 Conv. H-Bond/Asn181/3.36 Pi-Sigma/Ile335/3.53 Pi-Pi Stacked/Tyr407/4.84 Pi-Pi T-shaped/Phe208/4.55 Pi-Alkyl/Cys323/5.39	−8.8
Epigallocatechin	Conv. H-Bond/Cys323/3.58 Conv. H-Bond/Tyr444/2.28 Conv. H-Bond/FAD600/2.55 Conv. H-Bond/Thr336/2.27 Conv. H-Bond/Phe208/2.84 Conv. H-Bond/Asn181/2.64 Pi-Donor H-Bond/Tyr407/3.78 Pi-Donor H-Bond/Tyr444/3.74 Pi-Donor H-Bond/Tyr407/3.70 Pi-Sigma/Ile335/3.70 Pi-Sulfur/Cys323/4.65 Pi-Pi Stacked/Tyr407/4.40 Pi-Alkyl/Leu337/4.81	−7.2

**Table 2:** Peaks designation UPLC-ESI/MS of metabolites in *Tribulus terrestris* leaf extract.

No.	Formula	Transformations	Error (ppm)	[M-H]^−1^ Experimental	[M-H]^−1^ Theoretical	Identification
1	C_9_H_10_O_7_	Hydration, Oxidation	−0.03814	180.04225	179.03498	Caffeic acid
2	C_9_H_8_O_3_	Hydration, Oxidation	−0.02587	198.0402	199.0606	Hydroxycinnamic acid
3	C_21_H_20_O_11_	Reduction	−0.38036	196.05823	195.05095	Cynarosie
4	C_15_H_10_O_7_	Reduction	0.17143	301.0353	302.24	Quercetin
5	C_15_H_10_O_6_	Nitro Reduction	−0.21529	285.0409	286.24	Kaempferol
6	C_15_H_10_O_6_	Nitro Reduction	0.28338	285.04120	286.24	Luteoline
7	C_15_H_14_O_7_	Hydration, Oxidation	0.45039	356.07451	306.27	Epigallocatechin
8	C_21_H_20_O_10_	Reduction	0.18654	435.5	432.4	Apigetrin
9	C_27_H_30_O_16_	Hydratation	−1.45637	609.1482	610.1084	Rutin
10	C_45_H_72_O_17_	Reduction	−4.4	915.4550	915.4590	Terreside A
11	C_27_H_42_O_4_	Reduction	−0.38036	255.08642	430.30764	Hecogenine
12	C_39_H_62_O_14_	Desaturation, Nitro Reduction	0.3610	755.262	754.901	Terreside B
13	C_45_H_12_O_15_	Desaturation, Nitro Reduction	−0.38036	918.4232	915.4590	Terrestrosin C
14	C_39_H_62_O_13_	Desaturation, Nitro Reduction	0.95112	738.05805	738.05077	Trillarin
15	C_51_H_84_O_22_	Oxidation, Nitro Reduction	−0.10032	1047.5413	1049.2	Protodioscin
16	C_33_H_52_O_8_	Desaturation, Nitro Reduction	2.37949	560.22	576.761	Disogluside–Trillin

Adapted from https://doi.org/10.3390/ph17020200 by Bouabdallah et al., 2024 [35]. Pharmaceuticals. Copyright 2024 by Pharmaceuticals.

## Data Availability

The data presented in this study are available on request from the corresponding author.
